# Whole-genome sequencing and comparative genome analysis of *Xanthomonas fragariae* YM2 causing angular leaf spot disease in strawberry

**DOI:** 10.3389/fpls.2023.1267132

**Published:** 2023-12-18

**Authors:** Yue Qiu, Fangjun Wei, Han Meng, Menglin Peng, Jinhao Zhang, Yilu He, Lanfang Wei, Waqar Ahmed, Guanghai Ji

**Affiliations:** ^1^ State Key Laboratory for Conservation and Utilization of Bio-Resources in Yunnan, Yunnan Agricultural University, Kunming, Yunnan, China; ^2^ College of Agriculture, Anshun University, Anshun, Guizhou, China; ^3^ Key Laboratory of Agro-Biodiversity and Pest Management of Ministry of Education, Yunnan Agricultural University, Kunming, Yunnan, China; ^4^ Agricultural Foundation Experiment Teaching Center, Yunnan Agricultural University, Kunming, Yunnan, China; ^5^ College of Plant Protection, South China Agricultural University, Guangzhou, Guangdong, China

**Keywords:** *Xanthomonas fragariae*, collinearity analysis, functional annotation, virulence factors, genome comparison

## Abstract

**Background:**

Angular leaf spot disease caused by plant pathogenic bacterium *Xanthomonas fragariae* seriously threatens strawberry crop production globally.

**Methods:**

In this study, we sequenced the whole genome of *X. fragariae* YM2, isolated from Yunnan Province, China. In addition, we performed a comparative genome analysis of *X. fragariae* YM2 with two existing strains of *X. fragariae* YL19 and SHQP01 isolated from Liaoning and Shanghai, respectively.

**Results:**

The results of Nanopore sequencing showed that *X. fragariae* YM2 comprises one single chromosome with a contig size of 4,263,697 bp, one plasmid contig size of 0.39 Mb, a GC content ratio of 62.27%, and 3,958 predicted coding genes. The genome of YM2 comprises *gum*, *hrp*, *rpf*, and *xps* gene clusters and lipopolysaccharide (LPS), which are typical virulence factors in *Xanthomonas* species. By performing a comparative genomic analysis between *X. fragariae* strains YM2, YL19, and SHQP01, we found that strain YM2 is similar to YL19 and SHQP01 regarding genome size and GC contents. However, there are minor differences in the composition of major virulence factors and homologous gene clusters. Furthermore, the results of collinearity analysis demonstrated that YM2 has lower similarity and longer evolutionary distance with YL19 and SHQP01, but YL19 is more closely related to SHQP01.

**Conclusions:**

The availability of this high-quality genetic resource will serve as a basic tool for investigating the biology, molecular pathogenesis, and virulence of *X. fragariae* YM2. In addition, unraveling the potential vulnerabilities in its genetic makeup will aid in developing more effective disease suppression control measures.

## 1 Introduction

*Xanthomonas fragariae* is a Gram-negative plant pathogenic bacterium that causes angular leaf spot (ALS) in strawberries (Henry and Leveau, 2016). It is a distinct and homogeneous species in the genus *Xanthomonas* with a narrow host range, and only *Fragaria* spp. are its natural hosts (Vandroemme et al., 2013). The leaves and calyx of strawberry plants are particularly vulnerable to infection (Wang et al., 2018). *X. fragariae* enters leaf tissue through stomata and wounds, colonizing the intercellular spaces between mesophyll and vascular tissues and causing discrete angular lesions and tissue discoloration in strawberry leaves, ultimately leading to plant death (Wang and Turechek, 2020).

*X. fragariae* was declared a quarantine pathogen by the Mediterranean Plant Protection Organization, the European Union, and Korea (Kim et al., 2016a). In 1960, *X. fragariae* was initially reported in the United States (Kennedy and King, 1962). Since then, the bacterium has widely spread to other strawberry-producing areas due to human activities and the import of plant material (Gétaz et al., 2018). ALS is continuing to spread worldwide despite the efforts made by plant protection organizations to halt its dispersal (Bestfleisch et al., 2015). It has become an increasing threat and a major factor affecting strawberry production (Roberts et al., 1997) and responsible for main yield losses (Kim et al., 2016b). In case of severe infection, ALS reduces the yield and destroys the fruit quality, making it unsuitable for the market (Wang et al., 2018). In Tianjin, China, the incidence of ALS in strawberries was first reported in 2016 (Wang et al., 2017). Since then, this disease has been observed in multiple provinces of China, including Taiwan, Liaoning, Shanghai, and Yunnan (Wu et al., 2020; Feng et al., 2021; Song et al., 2021; Zhang et al., 2022). Therefore, ALS is a great concern to the strawberry industry and deserves more attention, as it spreads to new regions.

The development of DNA sequencing technology has led to an abundance of genomic data, providing us with the basic resources to fully explore the genetic diversity and pathogenic mechanism of a pathogen. The first draft genome of *X. fragariae* (LMG25863) was published in 2013, providing basic information about its biological characteristics and genetic information (Vandroemme et al., 2013). It was found that compared with other pathogenic *Xanthomonas* spp., *X. fragariae* has a narrow host range and a smaller genome and lacks some genes related to pathogenicity. However, it still contains major virulence-related gene regions. So far, 74 genome sequences of *X. fragariae* are available in the National Center for Biotechnology Information (NCBI) database, of which nine are complete genomes. As additional genome sequences emerge, we will better understand the genomic diversity and virulence variations among *X. fragariae* strains.

In September 2021, symptoms of ALS in strawberries were observed in the strawberry-growing regions of Yuxi and Kunming Cities, Yunnan Province, China (Zhang et al., 2022). Infected leaves were collected for bacterial isolation, and on Wilbrink-N agar medium plates, round, dense, and yellow color colonies were obtained (a typical feature of Xanthomonadaceae). According to Koch’s postulates, a pot experiment confirmed the pathogenicity of the isolated strain using the strawberry cultivar Monterey, and it was identified as *X. fragariae* YM2 based on molecular analysis (Zhang et al., 2022). This study aimed to sequence the whole genome of *X. fragariae* YM2 and to analyze its virulence-related gene content. In addition, a comparative genome analysis was performed with two existing strains of *X. fragariae* YL19 and SHQP01, isolated from Liaoning and Shanghai, respectively (Feng et al., 2021; Song et al., 2021). Our findings will serve as a useful tool for future research into *X. fragariae* virulence, particularly in elucidating its pathogenic mechanisms at the molecular level, which is essential for limiting its prevalence.

## 2 Materials and methods

### 2.1 Bacterial strains and growth medium condition

*X. fragariae* YM2 (accession no. OP847193) was previously isolated from the ALS-infected strawberry leaves collected from Yunnan Province, China (Zhang et al., 2022). The genome of *X. fragariae* YM2 was sequenced in this study, while genomes of *X. fragariae* YL19, *X. fragariae* SHQP01, and other relative strains were downloaded from the NCBI database (**Supplementary Table 1**). *X. fragariae* YM2 was grown on Wilbrink-N agar medium plates (proteose peptone 5 g/L, sucrose 10 g/L, MgSO_4_·7H_2_O 0.25 g/L, K_2_HPO_4_ 0.5 g/L, NaNO_3_ 0.25 g/L, agar 18 g/L, and pH 7.0) at 28°C for 72 h (Zhang et al., 2022). A 50% (v/v) glycerol solution was used to store pure cultures of bacterial strains at −80°C for later use.

### 2.2 DNA extraction, genome sequencing, and assembly

The total genomic DNA of *X. fragariae* YM2 was extracted from the overnight culture grown at 28°C and 160 rpm in Wilbrink-N broth using a QIAGEN Genomic-tip kit according to the manufacturer’s instructions (Liu et al., 2022). The genome was sequenced using the Nanopore PromethION platform at Biomarker Technologies (Beijing, China). The filtered reads were assembled using Canu (v1.5) software (Koren et al., 2017), and then the circlator (v1.5.5) was used to identify the circular sequence in the assembled genome. The complete genome sequence of *X. fragariae* YM2 was deposited in the NCBI (https://www.ncbi.nlm.nih.gov/) database under GenBank accession number CP114897.

### 2.3 Genome annotation

Prodigal (v2.6.3) software was used for coding gene prediction analysis (Hyatt et al., 2010). After masking putative functional genes, the GenBlastA (v1.0.4) tool scanned the complete genomes, and putative candidates were evaluated for non-mature and frame-shift mutations using GeneWise (v2.2.0). Transfer RNA (tRNA) gene prediction analysis was performed using tRNAscan-SE (v2.0) (Chan and Lowe, 2019), while ribosomal RNA (rRNA) gene prediction analysis was performed using Infernal (v1.1.3) (Nawrocki and Eddy, 2013). RepeatMasker was used to predict repetitive sequences. PhiSpy (v2.3) was used for prophage prediction, and CRT (v1.2) was employed for CRISPR identification. To predict genomic islands in the genome, IslandPath-DIMOB (v0.2) was utilized. The predicted proteins were blasted (e-value: 1e−5) against Nr, Swiss-Prot, TrEMBL, Kyoto Encyclopedia of Genes and Genomes (KEGG), eggNOG, and Blast2go for functional annotation. Based on the Comprehensive Antibiotic Research Database (CARD), RGI software was used to identify the corresponding sequences and to obtain antibiotic-resistant genes (Aziz et al., 2008). The genes related to virulence factors were annotated using the VFDB databases, and finally, the RAST server was used for fully automated annotation of the bacterial genomes (Aziz et al., 2008).

### 2.4 Phylogenetic relationship and average nucleotide identity calculation

Four housekeeping genes, including *atpD*, *dnaK*, *gyrB*, and *rpoD*, were chosen for multilocus sequence typing (MLST) analysis (Young et al., 2008; Parkinson et al., 2009; Bui Thi Ngoc et al., 2010). TBtools was used to obtain the sequences of these four genes from the genomes of *X. fragariae* YM2, which were then concatenated in the alphabetical order of the genes (Chen et al., 2020). Mega-X was used to perform sequence alignment, trimming, and phylogenetic analysis (Kumar et al., 2018). A multigene phylogenetic tree was constructed using the maximum likelihood method based on the four housekeeping gene concatenated sequences (Ahmed et al., 2022). JSpeciesWS was used to compute the average nucleotide consistency (ANI) analysis and digital DNA–DNA hybridization (dDDH) values (Richter et al., 2016). Genome-to-genome comparison was performed using the Genome-to-Genome Distance Calculator (GGDC; v3.0) according to default parameters (Meier-Kolthoff et al., 2013).

### 2.5 Comparative genomic analysis

The main virulence factors, orthologous genes, and genome collinearity analyses were performed to better understand the differences among *X. fragariae* strains YM2, YL19, and SHQP01. OrthoVenn online service was used to determine the Orthologous gene clusters (Wang et al., 2015), and TBtools (v1.120) was used to analyze the genomic synteny (Chen et al., 2020).

### 2.6 AntiSMASH analysis

AntiSMASH 5.0 with a web server was used to identify probable secondary metabolite biosynthesis gene clusters in the genomes of *X. fragariae* YM2, YL19, and SHQP01 (Blin et al., 2019). The GenBank databases were used to gain more detailed gene cluster information.

## 3 Results

### 3.1 Complete genome assembly and analysis

**Figure 1:**
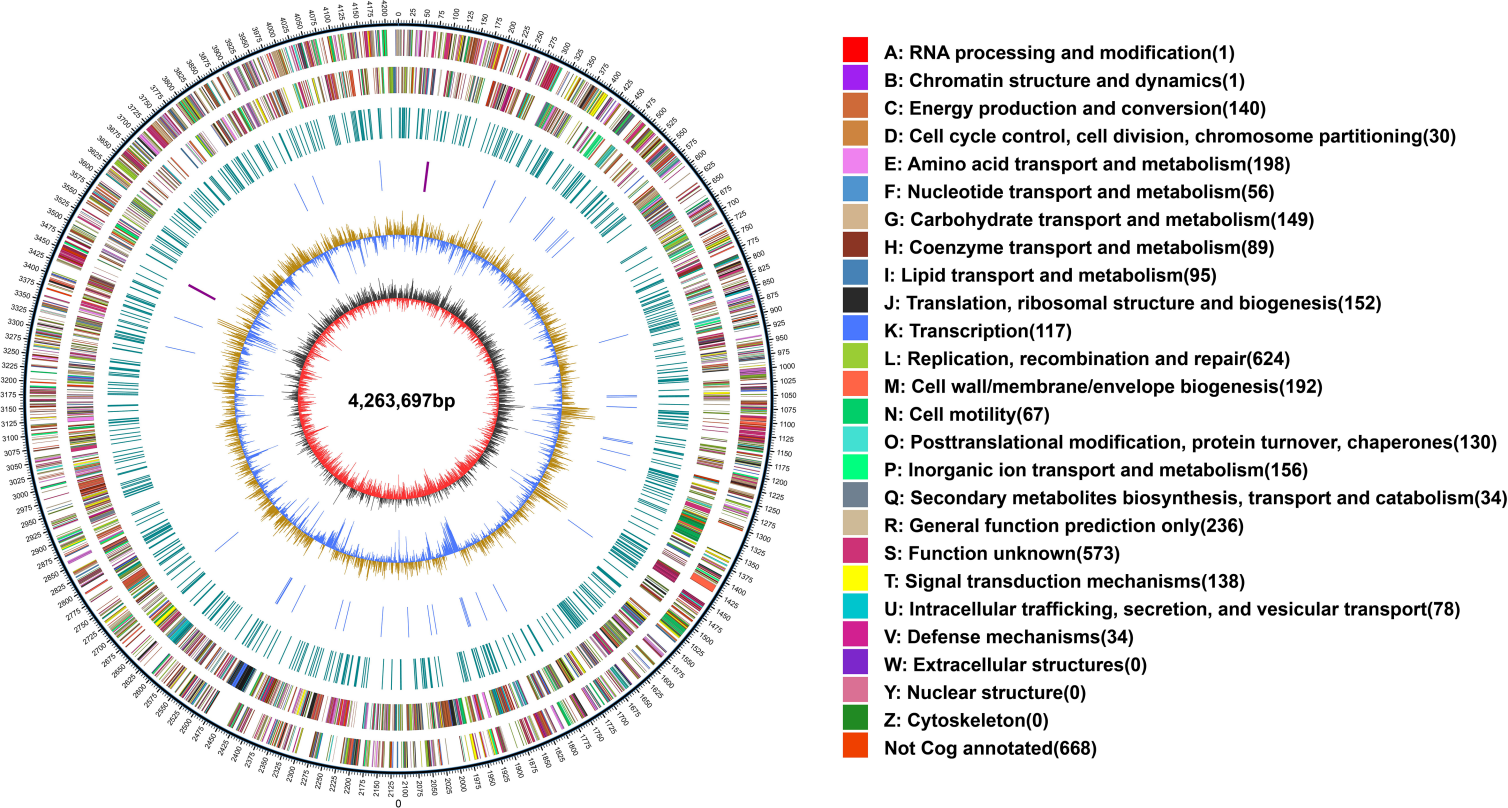
Whole-genome map of *Xanthomonas fragariae* YM2. The outermost circle is the mark of genome size, with each scale of 5 kb. The second and third circles are genes on the positive and negative chains of the genome, respectively. Different colors represent different COG functional classifications. The fourth circle is the repeat sequence. The fifth circle is tRNA and rRNA; blue is tRNA, and purple is rRNA. The sixth circle is the GC content. The innermost circle is GC skew. tRNA, transfer RNA; rRNA, ribosomal RNA.

The whole genome of *X. fragariae* YM2 was sequenced in this study, resulting in a total of 163,167 sequence reads with 1,441,130,510 bases. The final assembled genome comprised a single circular chromosome with an entire length of 4,263,697 bp and one plasmid contig (0.39 Mb) (**Figure 1**). The average GC content of the YM2 genome was found to be 62.27%, which is similar to YL19 (62.75%) and SHQP01 (62.0%) (**Table 1**). There were 3,958 coding genes with a length of 3,606,930 bp containing 59,507 bp repetitive sequences, accounting for 1.4% of genome sequences. Approximately 215 RNA-encoding genes were predicted, including six genes encoded for ribosomal RNAs, 54 encoded for transfer RNAs, and 155 identified as non-coding RNAs. The basic genome of YM2 was predicted to encode 20 pseudogenes with a length of 17,029 bp.

**Table 1:** The general genomic features of YM2, YL19, and SHQP01.

Parameters	YM2	YL19	SHQP01
Size of the assembled genome (Mb)	4.26	4.10	4.11
GC content (%)	62.27	62.75	62.0
Number of scaffolds	2	2	2
Number of genes	3,958	3929	3941
chromosome size (Mb)	4,22	4.09	4,09
Number of plasmids	1	1	1
Number of rRNA genes	6	6	6
Number of tRNA genes	54	54	54
Number of other ncRNA genes	155	254	233
Number of CRISPRs	9	2	2
Geographic location	Yunnan	Liaoning	Shanghai
Carbohydrate-active enzyme functional classification and corresponding genes			
Glycoside hydrolases	65	82	97
Glycosyl transferases	37	53	52
Polysaccharide lyases	6	3	3
Carbohydrate esterases	23	4	4
Auxiliary activities	6	2	1
Carbohydrate-binding modules	18	23	18
Total	155	167	175

rRNA, ribosomal RNA; tRNA, transfer RNA; ncRNA, non-coding RNA.

### 3.2 Gene prediction and annotation

**Figure 2:**
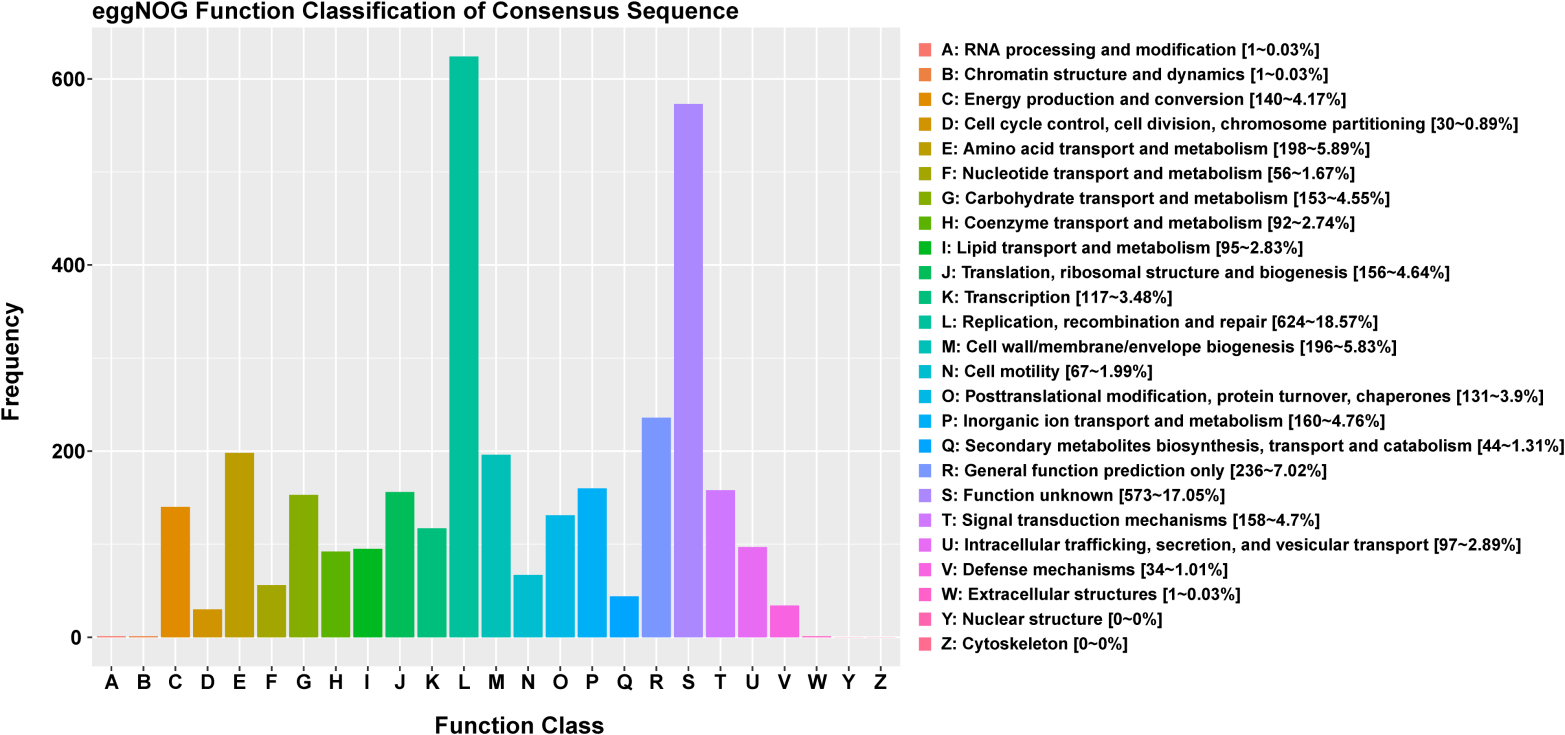
The eggNOG function classification of the consensus sequence.

**Figure 3:**
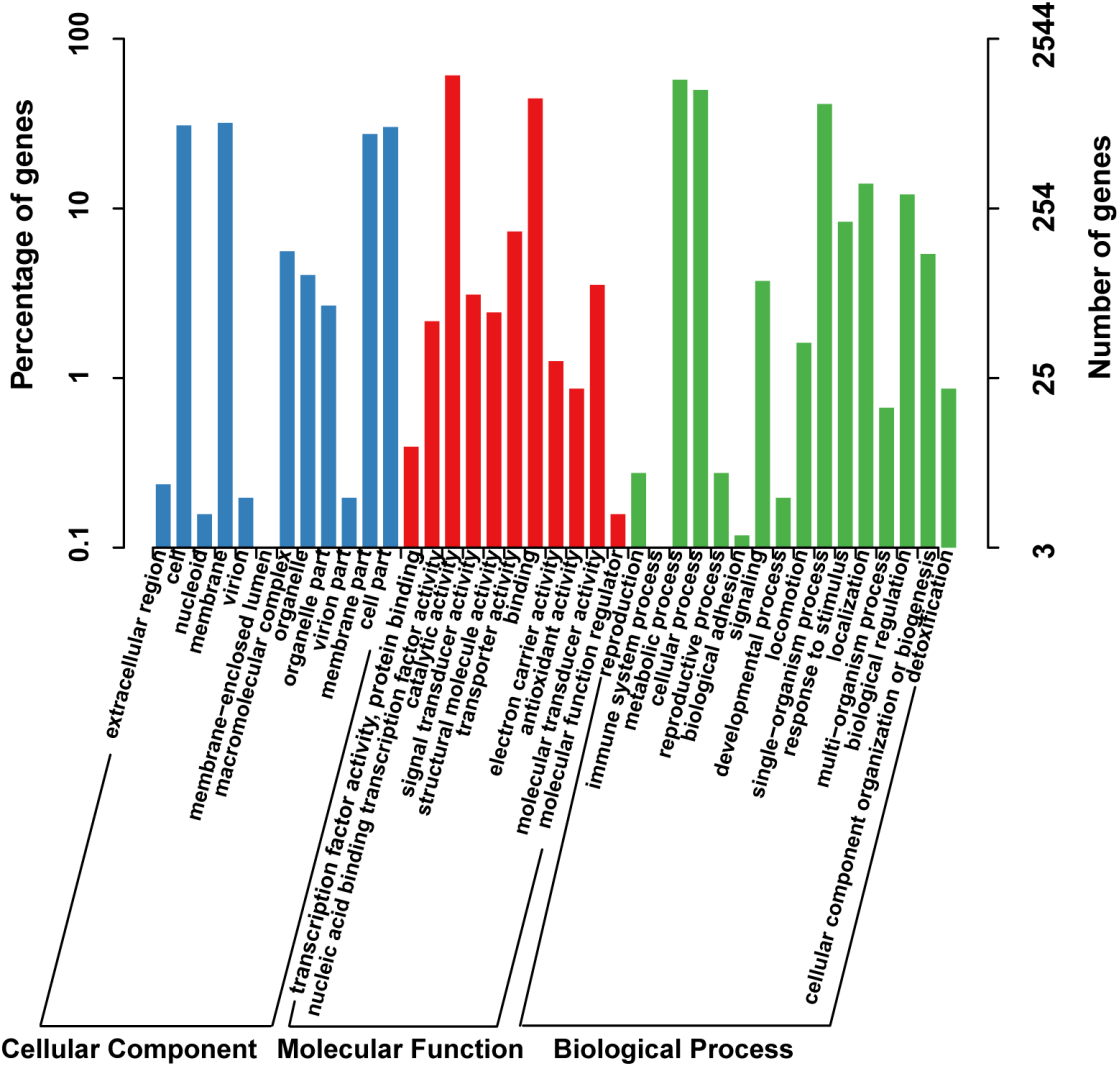
The GO function classification of the consensus sequence. GO, Gene Ontology.

**Figure 4:**
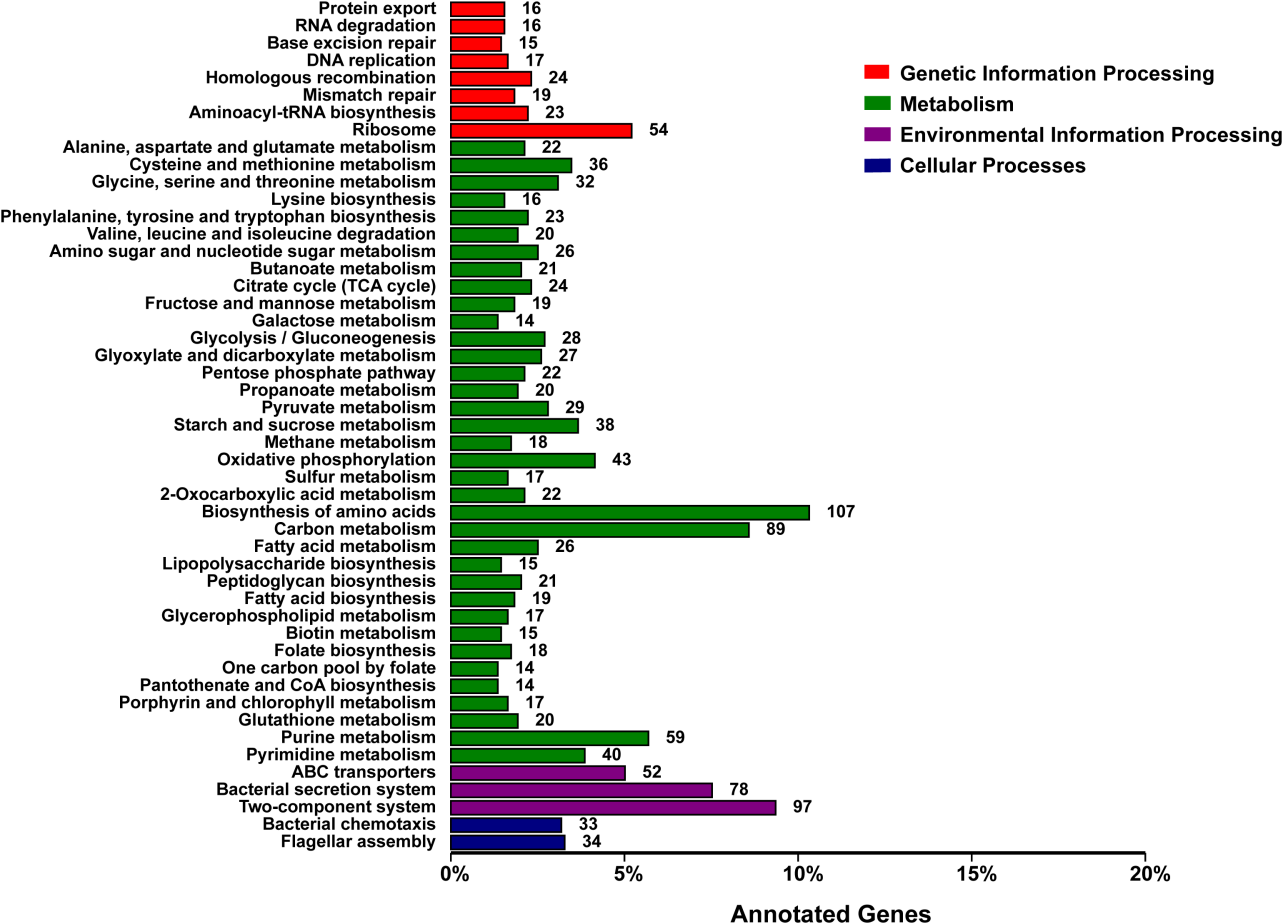
KEGG function classification of the consensus sequence. KEGG, Kyoto Encyclopedia of Genes and Genomes.

The functional classification of DNA coding sequences (CDSs) in YM2 was analyzed using several major databases, and the results showed that 3,936 genes were annotated as functional genes, accounting for 94.44% of the total genes (**Supplementary Table 2**). There were 3,289 protein-encoding genes in the eggNOG database, 2,543 in the Gene Ontology (GO) database, and 2,166 in the KEGG database. The eggNOG classification annotation showed that the genes for replication, recombination, and repair were the most abundant, with 624 genes accounting for 18.57%, followed by 573 genes of unknown function accounting for 17.05% (**Figure 2**). The GO classification annotation results indicated that 2,543 genes were annotated into 39 subclasses of three main categories: biological processes, cellular components, and molecular functions. Further analysis revealed that 38.39% of predicted genes were related to molecular function (mainly catalytic activity, binding, and transporter), 24.71% were involved in cellular components (mostly cell, cell part, membrane, and membrane part), and 36.9% were concentrated in biological processes (most of in metabolic process, cellular process, single organism process, and localization) (**Figure 3**). KEGG pathway enrichment analysis revealed that 2,166 genes were functionally annotated in 113 metabolic pathways (**Supplementary Table 3**). According to KEGG pathway annotation results, most genes were involved in four categories: genetic information process, metabolism, environmental information processing, and cellular processes (**Figure 4**). In the genetics information processing, most genes (54) were present in the ribosome (ko03010) pathway. The category metabolism contains the highest number of genes, among which 107 and 89 genes were involved in the biosynthesis of amino acid (ko01230) and carbon metabolism (ko01200) pathways, respectively. In environmental information processing and cellular processes, maximum genes of 97 and 34 were present in the two-component system (ko02020) and flagellar assembly (ko02040) pathways, respectively (**Figure 4**).

### 3.3 Phylogenetic relationship and average nucleotide identity calculation

**Figure 5:**
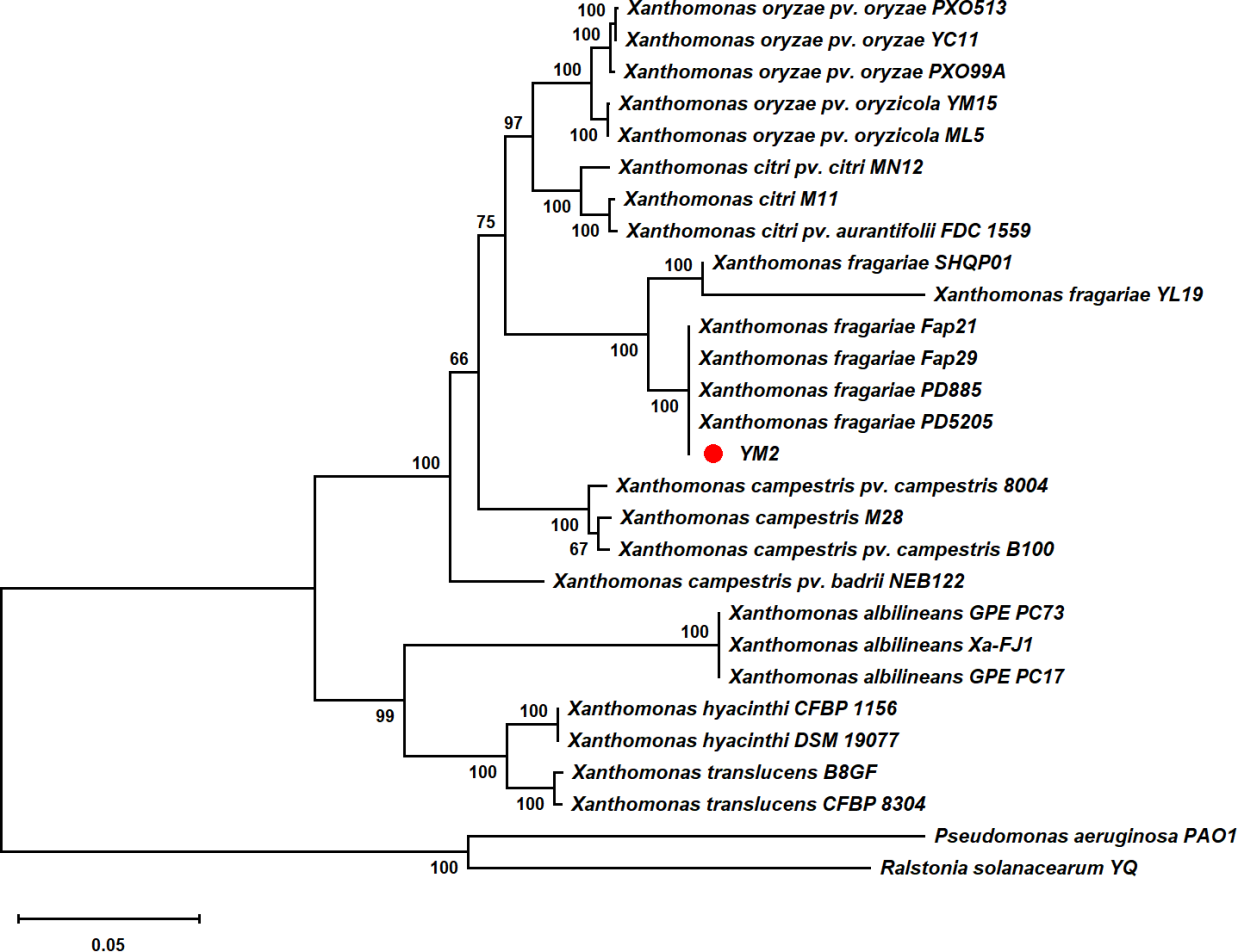
Maximum likelihood tree constructed with concatenated partial sequences of four housekeeping genes (*atpD*, *dnaK*, *gyrB*, and *rpoD*) of *Xanthomonas fragariae*. The tree scale represents 0.050.

Partial nucleotide sequences of four housekeeping genes (*atpD*, *dnaK*, *gyrB*, and *rpoD*) from YM2 and related species were aligned, trimmed, and concatenated to a final sequence of 7,656-bp nucleotide positions to infer a maximum likelihood phylogenetic tree. The dendrogram constructed using multilocus sequence analysis (MLSA) showed that strain YM2 was distributed in a separate branch along with other strains of *X. fragariae* Fap29, Fap21, PD8805, and PD5205, which represented a sister species with 100% bootstrap support value. In contrast, the strains YL19 and SHQP01 were located in another branch (**Figure 5**). ANI is mainly used to evaluate DNA similarity indexes between species at the genome-wide level. We conducted an ANI analysis between YM2 and six other *X. fragariae* strains present in the same clade in the phylogenetic tree. The results of the ANI analysis are consistent with the phylogenetic relationship (**Table 2**). YM2 has an ANI value of 94.69% with SHQP01 and 94.68% with YL19, slightly lower than the threshold of 95% for species differentiation. Meanwhile, the ANI value between strain YM2 and other *X. fragariae* strains (Fap29, Fap21, PD8805, and PD5205) was higher than 99% (**Table 2**). However, all the DNA–DNA hybridization (DDH) values among the strain sequenced in this study and those of the reference strains were consistently higher than 70.0% (**Table 2**). Based on all analytical results, the isolate YM2 causing ALS was identified as *X. fragariae*.

**Table 2:** ANI and dDDH values among the YM2 and other *Xanthomonas fragariae* strains.

Genome	Query genome of YM2	
	ANIb values [aligned nucleotides] (%)	DDH values (%)
*X. fragariae* FaP29	100.00 (98.90)	99.9
*X. fragariae* Fap21	100.00 (98.90)	99.9
*X. fragariae* PD5205	100.00 (98.90)	99.9
*X. fragariae* PD885	99.81 (98.24)	99.6
*X. fragariae* SHQP01	94.69 (76.97)	75.0
*X. fragariae* YL19	94.68 (77.00)	75.1

ANIb and DDH values indicate the pairwise comparisons of given genomic sequences with the genome of strain YM2.

ANI, average nucleotide consistency; dDDH, digital DNA–DNA hybridization.

### 3.4 Comparative genome analysis

**Figure 6:**
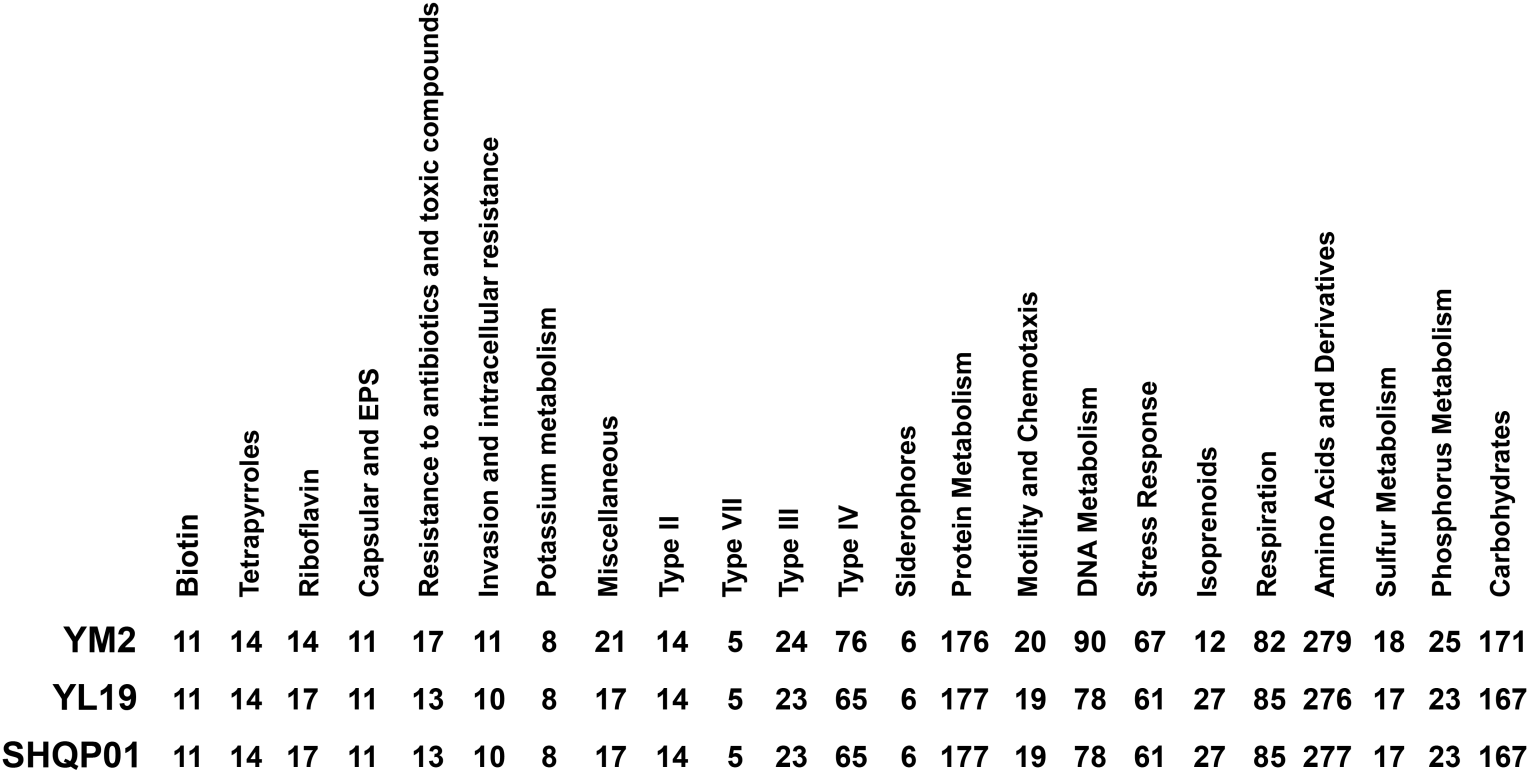
Comparison of genomic features of three *Xanthomonas fragariae* strains using the online annotation service RAST.

To comprehensively understand the distinctions between the three strains, YM2, YL19, and SHQP01, isolated from Yunnan, Liaoning, and Shanghai in China, respectively, we conducted a comparative investigation of their genome content and genome structure. The general genomic features of strains YM2, YL19, and SHQP01 are listed in **Table 1**. Genomic analysis revealed that YL19 is the most closely related to SHQP01 and the least closely related to YM2. Genome comparisons utilizing information from the RAST and SEED-Viewer databases revealed that the metabolic network and subsystem profiles of the YL19 and SHQP01 strains were nearly identical. In contrast, those of the YM2 strain differed slightly (**Figure 6**). The number of subsystems that are responsible for resistance to antibiotics and toxic compounds, Type IV (protein and nucleoprotein secretion system), DNA metabolism, stress response system, amino acids and derivatives, phosphorus metabolism, carbohydrates, and the miscellaneous subsystems in strain YM2 was higher than that in YL19 and SHQP01. The number of subsystems responsible for riboflavin biosynthesis, isoprenoid metabolism, and respiration subsystems in strain YM2 was lower than that in YL19 and SHQP01.

**Figure 7:**
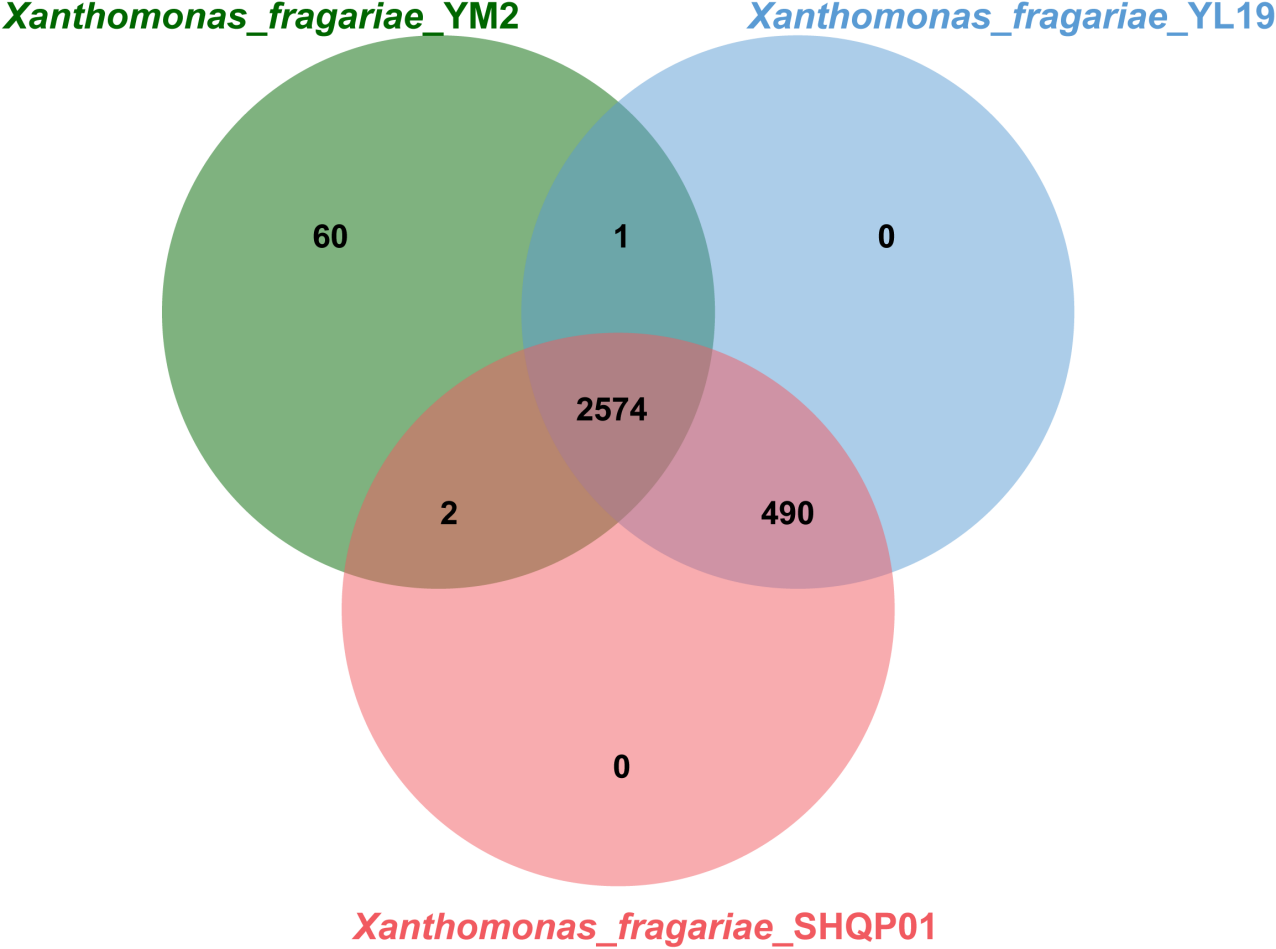
Venn diagram showing the shared orthologous clusters among the three *Xanthomonas fragariae* strains.

OrthoVenn online service determined the orthologous gene clusters among the three genomes. The results showed that 2,574 gene clusters containing 7,849 proteins were shared among the three strains (**Figure 7**). Furthermore, YL19 and SHQP01 shared 490 gene clusters but shared only one and two genes with YM2, respectively, suggesting that the genomic repertoires of YL19 and SHQP01 are closer. We found that strain YM2 contained 60 unique gene clusters containing 425 proteins, while no gene cluster and protein are unique to the YL19 and SHQP01 genomes (**Supplementary Table 4**).

### 3.5 Collinearity analysis

**Figure 8:**
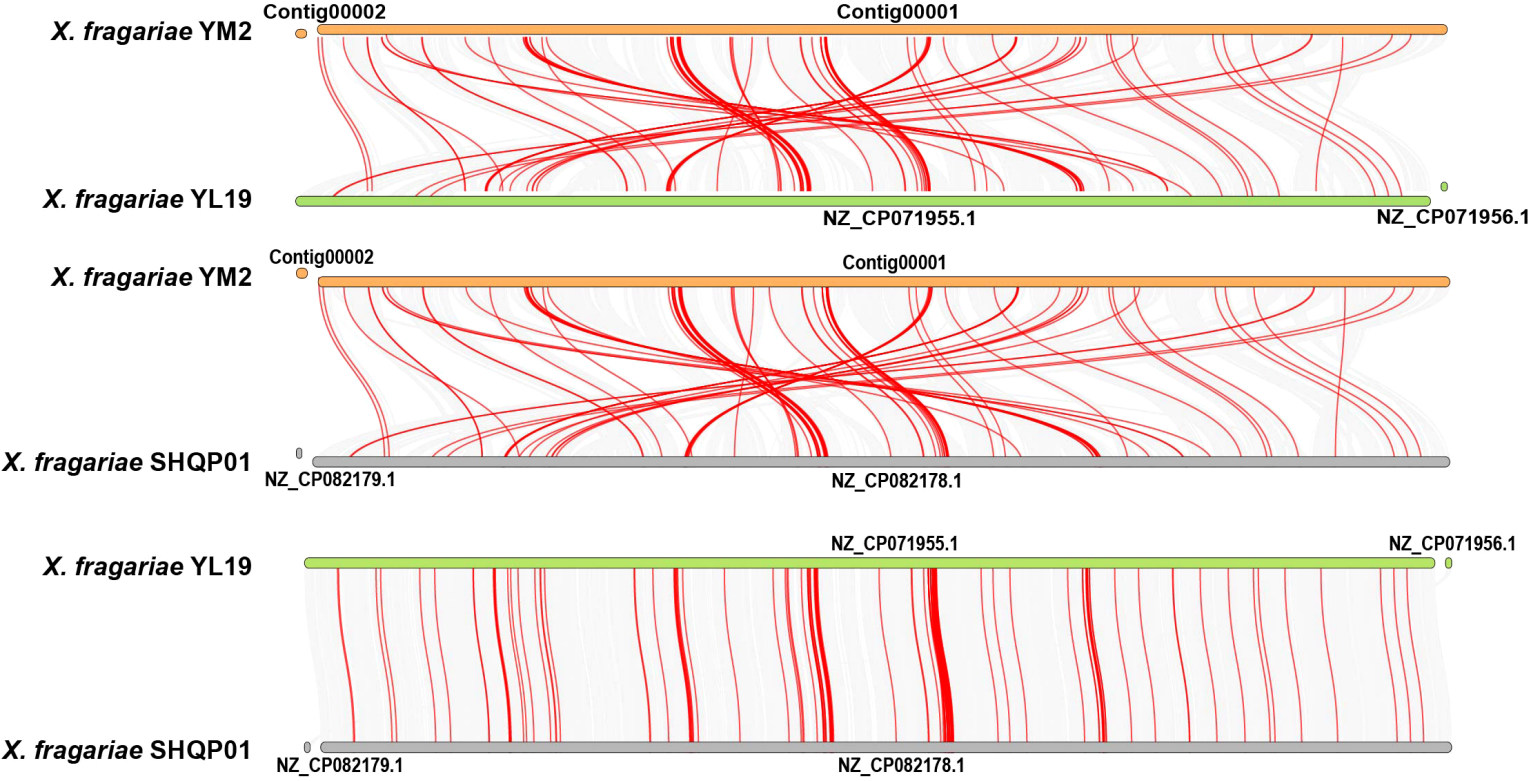
Synteny analyses of the genomes of three strains of *Xanthomonas fragariae*. Gray lines on the background indicate the collinear blocks between *X. fragariae* strains, and red lines highlight the syntenic major virulence-related gene pairs.

Collinearity analysis revealed a number of homologous regions in the YM2 and YL19 genomes, but some regions were rearranged and inverted (**Figure 8**). Similar results were found between the YM2 and SHQP01 genomes. The number of collinear genes between YM2 and YL19, and YM2 and SHQP01 was 2,705 and 2,701, respectively (**Supplementary Table 5**). YL19 and SHQP01 were the two strains with the most similar genome structure, with 3,614 collinear genes between them (**Figure 8**). The degree of collinearity between two species can be used to calculate their evolutionary distance. The better the genetic collinearity, the closer the evolutionary gap between species. Strain YM2 has lower similarity and longer evolutionary distance with strains YL19 and SHQP01, while YL19 has high similarity, good collinearity, and a higher degree of common origin compared with SHQP01 (**Figure 8**).

### 3.6 Pathogenicity-related genes in the genome of *X. fragariae* YM2

A total of 659 known or putatively linked genes encoding virulence factors were found in the genome of *X. fragariae* YM2. These virulence factors encode the genes related to carbohydrate-active enzymes (CAZymes), extracellular polysaccharides (EPSs) (encoding by *gum* gene cluster), lipopolysaccharide (LPS), type III secretion system (encoding by *hrp* gene cluster), T3SEs (encoding by *xop* gene cluster), type II secretion system (encoding by *xps* gene cluster), adhesins, methylase of chemotaxis methyl-accepting protein (encoding by *cheR4* gene), and others (**Supplementary Table 6**). Interestingly, *xcs* genes that code for T2SS were completely absent in the YM2 genome. In particular, no antibiotic-resistant genes were found in the YM2 genome after annotating via CARD.

### 3.7 Carbohydrate-active enzymes

Polysaccharides such as cellulose, hemicellulose, pectin, and lignin are woven to form the complex network of the plant cell wall. These polysaccharides are broken down by CAZymes, which provide nutrients to pathogenic bacteria. In our study, we conducted a comprehensive analysis of putative CAZymes, which revealed that the glycoside hydrolase (GH; 65 genes) family was the major category in the *X. fragariae* YM2 genome, followed by glycosyl transferases (GTs; 37 genes) and carbohydrate esterases (CEs; 23 genes). The total number of CAZyme genes (155) was lower relative to that in YL19 (167) and SHQP01 (175) (**Table 1**). According to the distribution of family and gene number in CAZyme classes, YL19 and SHQP01 are very similar, but YM2 is very different. The number of GHs and GTs in YM2 was lower than that in YL19 and SHQP01. In contrast, the number of CEs, auxiliary activities (AAs), and polysaccharide lyases (PLs) was higher in YM2 (**Table 1**).

### 3.8 Extracellular polysaccharide and lipopolysaccharide gene clusters

Many phytopathogenic bacteria produce large amounts of EPSs and LPSs during invasion in the host plants. The highly conserved *gum* gene cluster, which consists of 12 genes ranging from *gumB* to *gumM*, regulates EPS production (Lu et al., 2008). The YM2 genome possesses seven of 12 genes, including *gumB*, *gumC*, *gumD*, *gumF*, *gumG*, *gumJ*, and *gumM*. In addition, for the genes in the *rpf* gene cluster, which positively regulate the synthesis of extracellular enzymes, YM2 contains only four genes: *rpfE*, *rpfF*, *rpfG*, and *rpfN*. Except for *gumG* genes, which were not found in YL19 and SHQP01, the rest of *gum* genes and *rpf* genes had high similarity with the homologous genes in genomes of YL19 and SHQP01 (**Supplementary Table 6**). LPS is a component of bacterial cell surfaces that plants frequently identify as pathogen-associated molecular patterns (PAMPs). LPA comprises lipid A, core oligosaccharide, and O antigen polysaccharide encoded by *lpx*, *waa*, and *wb* gene clusters, respectively. Twenty-two genes associated with encoding LPS or LPS components were found in the genome of YM2, including *wzm* and *wzt* (**Supplementary Table 6**). However, three genes (*gtrB*, *wzm*, and *wzt*) out of 22 genes were not found in the YL19 and SHQP01 genomes. The other 19 genes were found to have higher homology in both the YL19 and SHQP01 genomes.

### 3.9 The *hrp* gene cluster

Plant pathogenic Gram-negative bacteria have evolved special export systems to target virulence factors in their hosts, and type III secretion system (TTSS) is one of them. The *hrp* gene cluster encoding TTSS in plant pathogens has been renamed as *hrp*, hrp-conserved (*hrc*), and *hrp*-associated (*hpa*) (Noël et al., 2002). The *hrp* gene cluster harbored in the YM2 genome contains 14 genes, including six *hpa* (*hpa1*, *hpa2*, *hpa3_1*, *hpa3_2*, *hpaB*, and *hpaP*), five *hrp* (*hrpB*, *hrpD6*, *hrpG*, *hrpW*, and *hrpX*), and three *hrc* (*hrcA*, *hrcR*, and *hrcS*). However, the genes coding for type III-secreted proteins HrpB2 and HrpF were absent.

### 3.10 Other pathogenicity-related gene cluster

More virulence and effector genes that influence host–pathogen interactions were investigated. In total, 1,168 PHI coding genes, 29.5% of the YM2 genome, were identified. Moreover, 367 secreted proteins, 367 signal peptides, and 813 transmembrane proteins were predicted. Two other genes, *rtxD* and *rtxE*, encoding repeat toxin (RTX) proteins with cytotoxic and hemolytic activity, were also present in the YM2 genome. In contrast, *rtxD* was absent in the genomes of YL19 and SHQP01. Iron is a necessary element for bacterial growth. Several gene clusters responsible for iron uptake and transport have been found in the YM2 genome: *feoA* and *feoB* are related to Fe^2+^ capture; nine *fepA* genes are responsible for transport siderophores; *fhuA* and *fhuE* are involved in the absorption of ferrichrome. All the iron uptake and transport genes were also present in the YL19 and SHQP01 genomes.

### 3.11 Secondary metabolite biosynthesis gene cluster

**Figure 9:**
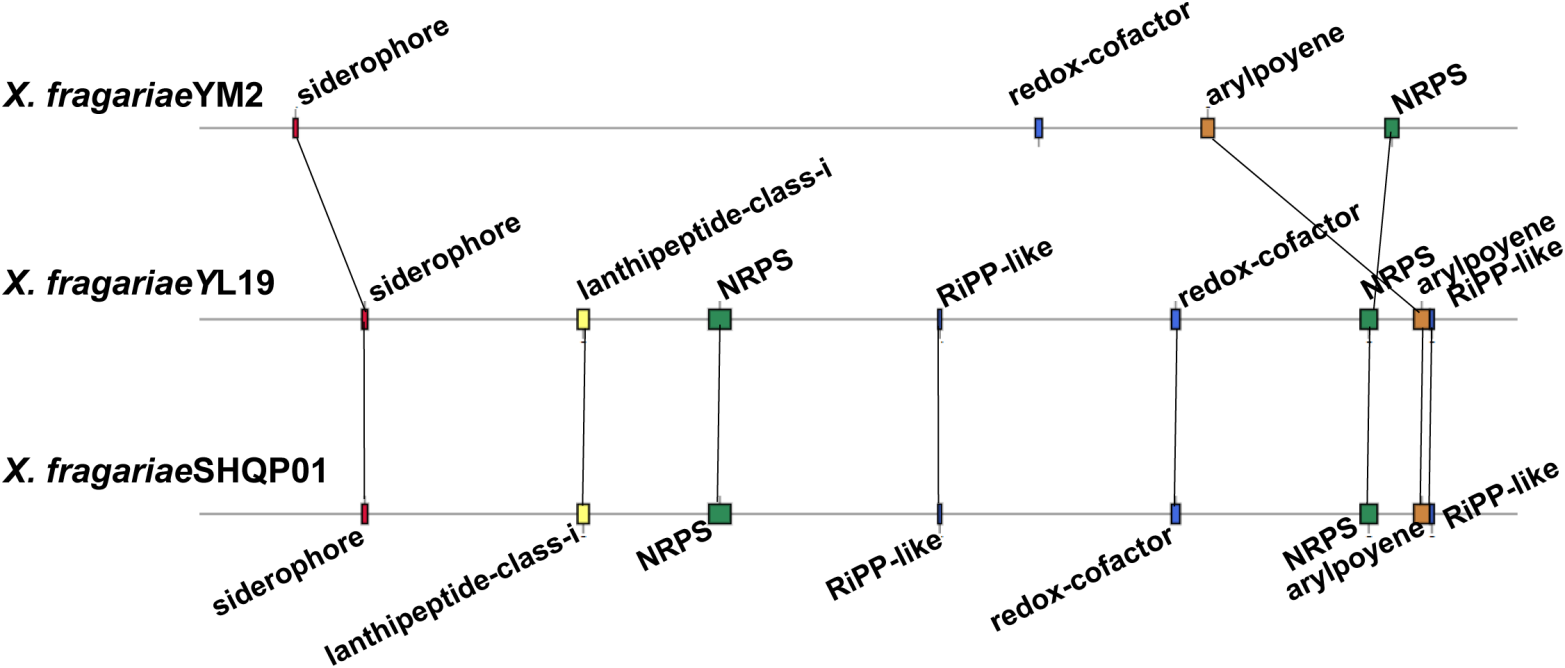
Comparison of the secondary metabolite biosynthesis gene clusters of *Xanthomonas fragariae* YM2 with *X. fragariae* YL19 and *X. fragariae* SHQP01. Black lines connect collinear regions.

The result of antiSMASH analysis showed that the genome of YM2 harbored four candidate gene clusters: siderophores, Redox-cofactor, arylpolyene, and non-ribosomal peptide synthetase (NRPS) (**Supplementary Table 7**). The first gene cluster was predicted to be the siderophore biosynthetic gene clusters associated with the biosynthesis of xanthoferrin. The arylpolyene cluster is associated with the biosynthesis of xanthomonadin, which plays an important role in epiphytic survival and host–pathogen interactions in the phytopathogenic *Xanthomonas* bacteria. However, the NRPS exhibited no similar gene cluster with the known strain. The genomes of YL19 and SHQP01 have the same number of secondary metabolic clusters, four more than YM2, including two other unspecified ribosomally synthesized and post-translationally modified peptide product (RiPP-like) clusters, and a cluster (lantipeptide class I) associated with glycopeptidolipid biosynthetic and an NRPS (**Figure 9**).

## 4 Discussion

Strawberry (*Fragaria × ananassa*) is one of the most popular cultivated fruits in the world because of its pleasing flavor and nutritional content (Zhang et al., 2011; Gétaz et al., 2020). To meet the domestic market demand, the cultivation area of strawberries has gradually expanded. China has become the world’s largest producer and consumer of strawberries, accounting for more than 50% of the world’s total output (Guan et al., 2022). However, strawberries are susceptible to various phytopathogenic organisms (Amil-Ruiz et al., 2011); one of the most important phytopathogenic organisms is *X. fragariae*. Once *X. fragariae* is allowed to multiply and expand in the field, it can devastate strawberry growth, cause yield loss, reduce quality, and make the fruit unmarketable (Wang et al., 2018). Moreover, the risk of ALS being introduced into new geographic areas is high because *X. fragariae* can persist in planting material for long periods asymptomatically. Many cases of intercepted infectious substances have confirmed the spread of this disease (Gétaz et al., 2018). Thus, to prevent the further spread of the disease, highly specific and effective detection is crucial. Further, the development of efficient disease management strategies is also needed to avoid significant economic losses.

*X. fragariae* can overwinter on old leaves or plant debris of strawberries, making it a source of infection in the coming year. The optimal temperature for growth of *X. fragariae* is ≈20°C, and the maximum is less than 32°C (Roberts et al., 1997). Thus, this bacterium grows well in the normal summer temperature in Yunnan Province, China. Therefore, there is a high probability of strawberry angular leaf spot outbreak during summer in Yunnan. Currently, no chemical or biological control agents effectively control the disease (Van Der Wolf et al., 2018). In addition, the excessive application of chemical pesticides can result in food safety concerns. Therefore, more studies are needed to assess its pathogenicity, genetic diversity, and control strategies to achieve maximum disease control while avoiding pesticide residues causing harm to humans.

Genome comparison is a valuable tool for studying the strain-to-strain variation in virulence, pathogenicity, and host fitness (Chen et al., 2022; Mafakheri et al., 2022). In this study, we sequenced the complete genome of *X. fragariae* YM2 and compared it with *X. fragariae* YL19 and *X. fragariae* SHQP01. The results showed that these three genomes were very similar in size, number of predicted genes, rRNAs, and tRNA genes but were significantly different in the number of other non-coding RNA (ncRNA) genes, CRISPRs, and CAZyme genes. Therefore, these differences may contribute to the differences in virulence between *X. fragariae* strains. The CRISPR–Cas system is an adaptive immune system that widely exists in prokaryotes and possesses resistance to foreign plasmids and phage sequences (Louwen et al., 2014). The current study found that YM2 contains more CRISPRs than YL19 and SHQP01; thus, we speculated that YM2 may have a stronger survival ability and may even be more difficult to control. CAZymes play an important role in plant pathogens’ invasion of plant tissues due to their ability to decompose plant cell walls and degrade macromolecular substances. The total number of CAZyme genes in YL19 is higher than that in YM2, suggesting that YL19 may have a stronger ability to decompose cell walls and stronger aggression than YM2. This result affirms the previous reports that YL19 not only causes ALS in strawberries but also causes crown infection pocket symptoms (Feng et al., 2021).

The three strains, YL19, SHQP01, and YM2, were chronologically isolated from Liaoning, Shanghai, and Yunnan, China, respectively. Interestingly, the MLSA, ANI analysis, and DDH analysis showed that YL19 and SHQP01 had a relatively close evolutionary relationship. At the same time, YM2 was more closely related to PD5205 (isolated in the Netherlands) and three other *X. fragariae* strains, PD885, Fap29, and Fap21 (all isolates of the USA) (Henry and Leveau, 2016). The ANI and DDH have been used as the standard for prokaryotic species classification at the genomic level when the ANI and DDH values between the compared strains were higher than the accepted thresholds defined for prokaryotic species, i.e., ANI > 95% and DDH > 70%, suggesting that the compared strains were the same species (Goris et al., 2007). Our results showed that YM2 has an ANI value of 94.69% with SHQP01 and 94.68% with YL19. These values are slightly lower than the cutoff score of 95%; however, according to the research of Konstantinidis and Tiedje (2005) and Richter and Rosselló-Móra (2009), this can still be classified as the same species. These results are consistent with Wei et al. (2023), who proved that YL19 and SHQP01 belong to the new subspecies *X. fragariae.*

Collinearity analysis is a standard investigative strategy used in comparative genomic studies to understand genomic conservation and the evolutionary relationships between multiple species (Wang et al., 2012). The results of genomic collinearity analysis suggest that YL19 and SHQP01 exhibit strong collinearity with each other. However, they both exhibit significant genome rearrangements in comparison to YM2. These differences may have led to the diversity of *X. fragariae*, which might be due to the evolution of the strains in different ecological environments. Compared with other pathogenic *Xanthomonas*, *X. fragariae* has a smaller genome and lacks some pathogenicity-related genes, which may explain why this bacterium is generally considered a mild pathogen. This is probably due to the protective mechanism of *X. fragariae* to ensure its long-term survival within the host.

Like *X. fragariae* LMG25863 (Vandroemme et al., 2013), YM2 also lacks genes coding for T2SS and *gum*-associated genes *gum*N, *gum*O, and *gum*P, but these genes were proved unnecessary for virulence of *Xanthomonas* spp. (Lu et al., 2008; Szczesny et al., 2010). The YM2 genome contains seven *gum* genes responsible for extracellular polysaccharide synthesis, *xps* genes for type II secretion, *hrp* genes for type III secretion, *rpf* genes for regulation of pathogenicity factors, and genes involved in the synthesis of lipopolysaccharide. These factors are considered the main pathogenicity/virulence factors in *Xanthomonas* spp. (An et al., 2020). Many previous studies have focused on exploring the functions of virulence factors. The deletion gene in the *gum* gene cluster reduced the production of xanthan gum (Vojnov et al., 2001) and biofilm formation (Rigano et al., 2007; Li and Wang, 2011). The *rpf* gene cluster regulates the synthesis of extracellular enzymes, extracellular polysaccharides, biofilm dispersal, and virulence (Dow et al., 2000; Dharmapuri et al., 2001). Similarly, RpfF directs the synthesis of DSF, and deletion of *rpfF* gene in *Xanthomonas oryzae* pv*. oryzae* leads to loss of virulence on rice (Chatterjee and Sonti, 2002), while disruption of *rpfG* reduces virulence in *Xanthomonas campestris* pv*. campestris* (Ryan et al., 2006). The genes *wzm* and *wzt* are predicted to encode components of an ABC transporter system to export LPS (Lu et al., 2008).

## 5 Conclusion

*X. fragariae* is classified as a quarantine pathogen and one of the most destructive pathogens of strawberries worldwide, particularly in China. In the study, we sequenced the complete genome of *X. fragariae* YM2; analyzed its genetic characteristics, taxonomic location, and virulence factors; and compared it with other *X. fragariae* strains, YL19 and SHQP01. According to the assembled and annotated results, we found 3,958 genes encoded by the YM2 genome with one chromosome. We observed that some gene families and clusters are important for the pathogenicity of *X. fragariae*. This in-depth study will serve as a great reference for future gene functional research and comparative genomic studies involving differences between species. Additionally, it will shed light on the pathogenicity of *X. fragariae* during host–pathogen interactions. Overall, the fully sequenced *X. fragariae* genome is a major step forward in bacterial genomics. However, future research on the construction of knock-out involving pathogenicity-related genes will provide new insights into the virulence of *X. fragariae* YM2 at the molecular level.

## Data availability statement

The datasets presented in this study can be found in online repositories. The names of the repository/repositories and accession number(s) can be found in the article/**Supplementary Material**. The complete genome sequences for YM2 have been deposited at GenBank under the accession number (CP114897).

## Author contributions

YQ: Data curation, Formal Analysis, Software, Writing – original draft. FW: Data curation, Formal Analysis, Writing – original draft. HM: Writing – Original draft, Formal Analysis, Software. MP: Data curation, Formal Analysis, Writing – original draft. JZ: Data curation, Investigation, Methodology, Writing – original draft. YH: Formal Analysis, Validation, Writing – original draft. LW: Conceptualization, Investigation, Writing – review & editing. WA: Supervision, Validation, Writing – original draft, Writing – review & editing. GJ: Project administration, Resources, Supervision, Writing – review & editing.
