# Supplementary material for: Whole-genome sequencing and comparative genome analysis of Xanthomonas fragariae YM2 causing angular leaf spot disease in strawberry
Source: Front Plant Sci. 2023 Dec 18;14:1267132. doi: 10.3389/fpls.2023.1267132 (PMC10773614; doi:10.3389/fpls.2023.1267132)
Supplement: Supplementary file 1 [file Table_1.docx]

| **Table S1. GenBank accession numbers of strains used in this study.** | | |
| --- | --- | --- |
| **Taxon** | **Strain** | **Accession number** |
| *Xanthomonas fragariae* | PD885 | NZ_LT853882 |
| *Xanthomonas fragariae* | PD5205 | NZ_LT853885 |
| *Xanthomonas fragariae* | FaP29 | NZ_CP016833 |
| *Xanthomonas fragariae* | FaP21 | NZ_CP016830 |
| *Xanthomonas fragariae* | YL19 | NZ_CP071955 |
| *Xanthomonas fragariae* | SHQP01 | NZ_CP082178 |
| *Xanthomonas fragariae* | YM2 | OP847193 |
| *Xanthomonas translucens* | B8GF | NZ_CP089999.1 |
| *Xanthomonas translucens* | CFBP 8304 | NZ_CP074365.1 |
| *Xanthomonas hyacinthi* | CFBP 1156 | NZ_CP043476.1 |
| *Xanthomonas hyacinthi* | DSM 19077 | NZ_JPLD01000001.1 |
| *Xanthomonas albilineans* | GPE PC73 | NC_013722.1 |
| *Xanthomonas albilineans* | GPE PC17 | NZ_JZIA01000003.1 |
| *Xanthomonas albilineans* | Xa-FJ1 | NZ_CP046570.1 |
| *Xanthomonas citri pv. aurantifolii* | FDC 1559 | NZ_CP011160.1 |
| *Xanthomonas citri pv. citri* | MN12 | NZ_CP008998.1 |
| *Xanthomonas citri* | M11 | NZ_CP029275.1 |
| *Xanthomonas campestris* | M28 | NZ_CP062066.1 |
| *Xanthomonas campestris pv. badrii* | NEB122 | NZ_CP051651.1 |
| *Xanthomonas campestris pv. campestris* | 8004 | NC_007086.1 |
| *Xanthomonas campestris pv. campestris* | B100 | NC_010688.1 |
| *Xanthomonas oryzae pv. oryzae* | YC11 | NZ_CP031464.1 |
| *Xanthomonas oryzae pv. oryzae* | PXO513 | NZ_CP033188.1 |
| *Xanthomonas oryzae pv. oryzae* | PXO99A | NC_010717.2 |
| *Xanthomonas oryzae pv. oryzicola* | YM15 | NZ_CP007810.1 |
| *Xanthomonas oryzae pv. oryzicola* | ML5 | NZ_CP059710.1 |
| *Pseudomonas aeruginosa* | PAO1 | NC_002516.2 |
| *Ralstonia solanacearum* | YQ | NZ_CP059489.1 |
