# Supplementary material for: Whole-genome sequencing and comparative genome analysis of Xanthomonas fragariae YM2 causing angular leaf spot disease in strawberry
Source: Front Plant Sci. 2023 Dec 18;14:1267132. doi: 10.3389/fpls.2023.1267132 (PMC10773614; doi:10.3389/fpls.2023.1267132)
Supplement: Supplementary file 2 [file Table_2.docx]

| **Table S2. The statistics result of CDS annotation.** | | |
| --- | --- | --- |
| **Database** | **Number** | **Percentage (%)** |
| eggNOG_Annotation | 3289 | 83.10% |
| GO_Annotation | 2543 | 64.25% |
| kegg_Annotation | 2166 | 54.72% |
| nr_Annotation | 3935 | 99.42% |
| Pfam_Annotation | 3095 | 78.20% |
| Swissprot_Annotation | 2048 | 51.74% |
| TrEMBL_Annotation | 2048 | 51.74% |
| All_Annotated | 3936 | 99.44% |
