# Supplementary material for: Whole-genome sequencing and comparative genome analysis of Xanthomonas fragariae YM2 causing angular leaf spot disease in strawberry
Source: Front Plant Sci. 2023 Dec 18;14:1267132. doi: 10.3389/fpls.2023.1267132 (PMC10773614; doi:10.3389/fpls.2023.1267132)
Supplement: Supplementary file 6 [file Table_6.docx]

| **Table S6. Major virulence related genes in the YM2 genome and the homology of genes in the YL19 and SHQP01.** | | | | | |
| --- | --- | --- | --- | --- | --- |
| **Gene Cluster** | **Gene name** | **Gene ID** | **Gene function** | **YL19** | **SHQP01** |
| *gum* | *gumB* | GE002108 | Protein GumB | 96.26% | 96.26% |
|  | *gumC* | GE002107 | Xanthan chain-length determinant | 97.12% | 97.12% |
|  | *gumD* | GE002106 | Protein GumD | 96.84% | 96.84% |
|  | *gumF* | GE002104 | Protein GumF | 80.77% | 80.77% |
|  | *gumG* | GE002103 | Xanthan biosynthesis acetyltransferase GumG | Non | Non |
|  | *gumJ* | GE002100 | Protein GumJ | 87.38% | 87.38% |
|  | *gumM* | GE002097 | GumM protein | 93.08% | 93.08% |
| LPS | *acpXL* | GE000508 | acyl carrier protein | 99.17% | 99.17% |
|  | *bplA* | GE002361 | probable oxidoreductase | 96.76% | 96.76% |
|  | *bplA* | GE002761 | probable oxidoreductase | 96.60% | 96.60% |
|  | *fabZ* | GE002040 | (3R)-hydroxymyristoyl ACP dehydratase | 96.97% | 96.97% |
|  | *glf* | GE003794 | UDP-galactopyranose mutase | 97.66% | 97.66% |
|  | *gluP* | GE001672 | glucose/galactose transporter | 97.79% | 97.79% |
|  | *gluP* | GE002744 | glucose/galactose transporter | 97.97% | 97.97% |
|  | *gluP* | GE003123 | glucose/galactose transporter | 96.61% | 96.61% |
|  | *gtrB* | GE000195 | bactoprenol glucosyl transferase | Non | Non |
|  | *gtrB* | GE002159 | bactoprenol glucosyl transferase | 96.87% | 96.87% |
|  | *gtrB* | GE003544 | bactoprenol glucosyl transferase | 98.20% | 98.20% |
|  | *kdsA* | GE001562 | 2-dehydro-3-deoxyphosphooctonate aldolase | 97.60% | 97.60% |
|  | *kdtB* | GE000781 | lipopolysaccharide core biosynthesis protein | 99.01% | 99.01% |
|  | *lpsB/lpcC* | GE002102 | Lipopolysaccharide core biosynthesis mannosyltransferase LpcC | 86.20% | 86.20% |
|  | *manAoAg* | GE001670 | mannose-6-phosphate isomerase | 95.69% | 95.69% |
|  | *waaA* | GE002673 | lipopolysaccharide core biosynthesis protein WaaP | 96.55% | 96.55% |
|  | *wbkC* | GE000585 | GDP-mannose 4,6-dehydratase / GDP-4-amino-4,6-dideoxy-D-mannose formyltransferase | 97.16% | 97.16% |
|  | *wbkC* | GE003147 | GDP-mannose 4,6-dehydratase / GDP-4-amino-4,6-dideoxy-D-mannose formyltransferase | 97.62% | 97.62% |
|  | *wbpL* | GE001503 | undecaprenyl-phosphate alpha-N-acetylglucosaminyltransferase | 97.32% | 97.32% |
|  | *wbpL* | GE002576 | undecaprenyl-phosphate alpha-N-acetylglucosaminyltransferase | 97.51% | 97.51% |
|  | *wzm* | GE000184 | lipopolysaccharide O-antigen ABC transport system transmembrane component | Non | Non |
|  | *wzt* | GE000185 | lipopolysaccharide O-antigen ABC transport system ATP-binding component | Non | Non |
| *hrp* | *hpa1* | GE000742 | Hpa1 protein | 85.61% | 85.61% |
|  | *hpa2* | GE000743 | Type III secretion-system related transglycosylase | 97.03% | 97.03% |
|  | *hpa3_1* | GE002653 | Hpa3 protein, type III secretion system | 95.83% | 95.83% |
|  | *hpa3_2* | GE000718 | Hpa3 protein, type III secretion system | 92.12% | 92.12% |
|  | *hpaB* | GE000722 | HpaB protein, type III secretion system | 97.67% | 97.67% |
|  | *hpaP* | GE000730 | Type III secretion control protein HpaP | 95.28% | 95.28% |
|  | *hrcA* | GE001715 | Heat-inducible transcription repressor HrcA | 97.34% | 97.34% |
|  | *hrcR* | GE000728 | Type III secretion system protein | 97.36% | 97.36% |
|  | *hrcS* | GE000727 | HrcS homolog | 98.08% | 98.08% |
|  | *hrpB* | GE000016 | ATP-dependent helicase HrpB | 96.84% | 96.84% |
|  | *hrpD6* | GE000724 | Protein HrpD6 | 95.47% | 95.47% |
|  | *hrpG* | GE000367 | HrpG protein | 98.22% | 98.22% |
|  | *hrpW* | GE000721 | HrpW protein | 91.04% | 91.04% |
|  | *hrpX* | GE000368 | HrpX protein | 97.90% | 97.90% |
| *xop* | *xopAD_1* | GE002900 | Type III effector protein XopAD | 94.15% | 94.15% |
|  | *xopAD_2* | GE002847 | Type III effector protein XopAD | 91.18% | 91.18% |
|  | *xopAD_3* | GE002059 | Type III effector protein XopAD | 91.32% | 91.32% |
|  | *xopAD_4* | GE001198 | Type III effector protein XopAD | 89.98% | 89.98% |
|  | *xopAD_5* | GE000085 | Type III effector protein XopAD | 90.59% | 90.59% |
|  | *xopAF* | GE000236 | Type III effector protein XopAF | 89.49% | 89.49% |
|  | *xopF1_1* | GE002652 | Type III effector protein XopF1 | 90.77% | 90.77% |
|  | *xopF1_2* | GE000719 | Type III effector protein XopF1 | 93.65% | 93.65% |
|  | *xopP_1* | GE003453 | Type III effector protein XopP | 89.02% | 89.02% |
|  | *xopP_2* | GE003452 | Type III effector protein XopP | 88.97% | 88.97% |
|  | *xopP_3* | GE003451 | Type III effector protein XopP | 88.53% | 88.53% |
|  | *xopP_4* | GE003450 | Type III effector protein XopP | 88.36% | 88.36% |
|  | *xopP_5* | GE003449 | Type III effector protein XopP | 87.81% | 87.81% |
|  | *xopR* | GE000003 | Type III effector protein XopR | 89.32% | 89.32% |
|  | *xopV* | GE002835 | Type III effector protein XopV | 89.64% | 89.64% |
| *xps* | *xpsD* | GE002446 | General secretion pathway protein D | 89.99% | 89.99% |
|  | *xpsG* | GE002454 | Type II secretory pathway pseudopilin | 98.15% | 98.15% |
|  | *xpsK* | GE002450 | Type II secretion system protein K | 97.3% | 97.3% |
|  | *xpsL* | GE002449 | General secretion pathway protein L | 96.88% | 96.88% |
|  | *xpsM* | GE002448 | General secretion pathway protein M | 97.86% | 97.86% |
| *rpf* | *rpfE* | GE001429 | Regulatory protein RpfE | 97.29% | 97.29% |
|  | *rpfF* | GE001422 | Enoyl-CoA hydratase | 97.13% | 97.13% |
|  | *rpfG* | GE001425 | Response regulator | 97.98% | 97.98% |
|  | *rpfN* | GE000798 | Porin | 96.15% | 96.15% |
| iron uptake and transport | *feoA* | GE001440 | Ferrous iron uptake protein | 95.53% | 95.53% |
|  | *feoB* | GE001439 | Ferrous iron transport protein B | 97.69% | 97.69% |
|  | *fepA* | GE000409 | outer membrane receptor FepA | 96.21% | 96.21% |
|  | *fepA* | GE000410 | outer membrane receptor FepA | 97.82% | 97.82% |
|  | *fepA* | GE002055 | outer membrane receptor FepA | 91.17% | 91.17% |
|  | *fepA* | GE002702 | outer membrane receptor FepA | 97.00% | 97.00% |
|  | *fepA* | GE003134 | outer membrane receptor FepA | 96.47% | 96.47% |
|  | *fepA* | GE000412 | ferrienterobactin outer membrane transporter | 96.90% | 96.90% |
|  | *fepA* | GE002527 | ferrienterobactin outer membrane transporter | 97.21% | 97.21% |
|  | *fepA* | GE002647 | ferrienterobactin outer membrane transporter | 94.44% | 94.44% |
|  | *fepA* | GE002665 | ferrienterobactin outer membrane transporter | 94.97% | 94.97% |
|  | *fepC* | GE003775 | iron-enterobactin transporter ATP-binding protein | 96.35% | 96.35% |
|  | *fhuA_1* | GE000685 | TonB-dependent receptor | 90.85% | 90.85% |
|  | *fhuA_2* | GE000684 | TonB-dependent receptor | 84.28% | 84.28% |
|  | *fhuA_3* | GE000601 | TonB-dependent receptor | 96.89% | 96.89% |
|  | *fhuE* | GE003835 | Ferric iron uptake outer membrane protein | 91.97% | 91.97% |
| *rtx* | *rtxD* | GE002218 | RTX toxin transporter, transmembrane protein linking with the outer membrane porin TolC | Non | Non |
|  | *rtxE* | GE001702 | RTX toxin transporter, ATPase protein | 97.12% | 97.12% |
| Non indicated that the gene product was not identified. | | | | | |
