# Supplementary material for: Whole-genome sequencing and comparative genome analysis of Xanthomonas fragariae YM2 causing angular leaf spot disease in strawberry
Source: Front Plant Sci. 2023 Dec 18;14:1267132. doi: 10.3389/fpls.2023.1267132 (PMC10773614; doi:10.3389/fpls.2023.1267132)
Supplement: Supplementary file 7 [file Table_7.docx]

| **Table S7**. **Secondary metabolite biosynthesis gene clusters of YM2 predicted by antiSMASH** | | | |
| --- | --- | --- | --- |
| **Cluster** | **Type** | **Location** | **Most similar known cluster (%)**^a^ |
| r1c1 | siderophores | 306,749-321,419 | xanthoferrin biosynthetic gene cluster (100%) |
| r1c2 | Redox-cofactor | 2,676,640-2,698,791 | lankacidin C biosynthetic gene cluster (13%) |
| r1c3 | arylpolyene | 3,206,647-3,247,843 | xanthomonadin I biosynthetic gene cluster (71%) |
| r1c4 | NRPS | 3,793,573-3,837,559 | ND |
| ^a^ indicates sequence similarity of gene cluster between *YM2* and the best BLAST hit from the antiSMASH database.  ND indicates that similar gene cluster was not detected in the antiSMASH database. | | | |
